# Extracts and Gleanings

**Published:** 1876-10

**Authors:** 


### Extracts and Gleanings.

*Iodoform as a Topical Application, Particularly in Venerea Diseases.* — MM. Dubrisay and Pelletan, in independent brouchers upon this subject, arrive at the following conclusions in common (*Jour de Dermatol, et Syph.*):

1. Iodoform is a local anæsthetic.

2. Applied in the form of a powder it cicatrizes wounds rapidly.

3. It is especially indicated in small superficial atonic wounds, or those having a tendency to phagedæna, soft chancres, suppurating buboes, syphilitic onychia, syphilides generally, varicose, scrofulous and cancerous ulcers.

4. It operates more surely and promptly than other therapeutic agents ordinarily employed in the cicatrization of ulcerating syphilides, under whatever form they may present themselves.

5. In the treatment of soft chancre, it is in some sort a specific in the promptitude with which it causes cicatrization without pain.

6. In the treatment of simple or virulent buboes (non-specific) it may be employed in the form of ointment as a re olvent during the first period with more success than a blister or tincture of iodine. During the period which succeeds the opening of the sore, it hastens rapid cicatrization of the wound.

7. In cases of soft chancre, of ulcerating syphilides and of bubo, when the suppuration is abundant, it is preferable to commence the treatment by solution of iodoform in glycerine and alcohol. The iodoform in powder may be used later.

8. The employment of iodoform in syphilitic affections does not do away with the necessity of using internal treatment.

9. The rapid cicitrazation brought about by iodoform is due— 1, to the simplicity of the dressing, which does not irritate the diseased parts; 2, to absorption of the secretions of the powder; 3, to its anticeptic properties, particularly when it is dissolved in glycerine and alcohol; 4, to the presence of iodide, which acts favorably on syphilitic ulcerations of all kinds.

SOLUTION OF IODOFORM.

R. Iodoform........................ oz. i to oz. iss
Glycerine..............................f. oz. xij
Alcohol ................................f. oz. iv.
M. Ft. sol.

IODOFORM OINTMENT.

R. Iodoform...........................grs. xxx
Alcohol.........................................q. s
Axungiac................. ..............oz i
M. Ft. unguent.

*To Prevent the Secretion of Milk in the Female Breast.*—I have for more than ten years employed the following method to prevent the secretion of milk in the breasts of women who may have had still-born children, or who, after having nursed their child for a few months, found it necsssary to wean it. It is perfectly clean and painless as far as my experience goes, and as such I beg to recommend it to the notice of my medical brethren.

We will take, for instance, the case where the infant has been born at the full period, but is dead, or dies within a few hours of its birth. The milk makes its appearance in the breasts generally about the second day, sometimes longer, and sometimes it is ready when the child is born, and in the case of still-born children, my experience leads me to think that in such cases, it makes its appearance earlier than when the child is born alive. My plan consists in taking a piece of emplastrum adhæsivum of about ten inches square, round the corners, cut a hole in the centre for the nipple, then from the centre of each corner make a straight cut towards and within two inches of the centre hole ; having now got it ready, let the patient lie on her back, her body being perfectly horizontal; warm the plaster and place it over the breast, then strap one of the lower corners down first, draw the opposite one tightly upwards and fix it in its place, then the other lower corner, and lastly the opposite upper one, having drawn it sufficiently tight first; now take a piece of plaster two inches wide and about sixteen or eighteen inches long, and put it on from below and outside the breast, across close by inside of nipple, and fasten the end over the clavicle; another piece may also be put on in an opposite direction, it being drawn over the shoulder. Of course, in cutting the plaster and strips, the size of the breasts must be taken into consideration, there being so much difference in the size of female breasts.

The above plan I always follow when one of my patients wishes to dry the milk, as they usually call it, or when they are compelled to do so either from the death of the ch ld or any other cause. I also am certain strapping will prevent mammary abscess if resorted to in the earlier stage; I, at least, have found it do so in many cases.—*Dr. J. W. Lane, in Medical Press and Circular.*

*Typhoid Fever.*—Dr. Crawford reports four series, of ten cases each, of typhoid fever. The first series was treated with alteratives, turpentine, egg-nog, dieuretics, with leeches, sponging, etc., lecally. There were six deaths. In the second series the treatment was stimulants, nutritious diet and Dover powder. Three deaths resulted. In the third group the treatment consisted in alteratives, anodynes, stimulants, nutritious diet, strychnia. nitric acid, turpentine emulsions and diuretics, Two cases were fatal. None of the treatment in any of the preceding cases have had the slightest effect in lessening the "dead epithelium" on the "surface of the digestive tube." The fourth group of cases was treated with dilte muriatic acid, beef tea and milk, with recovery in every case. In all these ten cases there was an absence of diarrhœa, suppression of urine, pneumonia complications, and indeed all other complications. The tongue was cleaned in an average of fifty-one hours from the commencement of the acid treatment, and with one or two exceptions remained clean during the course of the disease.

He believes quinine aggravates the disease in all cases uncomplicated with malaria. He avers that in two cases where, during the second week of the disease, the tongue was clean and moist, he tried the experiment of giving half grain doses of *quinia Sulphas.* The result was that in both cases a "dry and irritable condition of the tongue" was induced within twenty-four hours. He believes that muriatic acid is "*the best* remedy known" for typhoid fever.—*Chicago Mediacl Journal,* March, 1875.

*Permanganate of Potash in Leucorrhœa.*—Dr. H. Fitzpatrick, (*Nashville Jourual of Medicine and Surgery,*) says: Some three years ago, I noticed permanganate of potassa recommended in gonorrhœa. As I was treating a very obstinate and troublesome case at the time, I determined to give it a trial.

The medicine succeeded so admirably, that I have never used any other medicine as an injection since, as none cther has succeeded so well in my hands.

Reasoning from analogy, I concluded that the permanganate would be good in leucorrhœa, also, (I have not seen it recommended,) and soon after gave it a trial, in the same manner in which I had used it in gonorrhœa—about three grains to the ounce of water, as an injection, twice a day, and with equally good results.

Of course the general health thould be attended to. I generally succeed now, in cases of gonorrhœa, with the internal use of sweet spirits nitre and bal. cop., with injections of the permanganate.

In leucorrhœa, I use the following pill, when the bowels are constipated:

R. Rhei pulvis............................

Castile soap..............................*aa* dr.j

Podophylin.............................. gr. xx

Make it into twenty pills, one to be taken at bed time, as often as it is necessary to keep the bowels soluble, and give Mur. Tinct. ferri three times a day, with injections of the permanganate twice a day. In some nervous cases I have used Hall's solution of strychnia, instead of the pill and iron, with good results.

In this manner, I have succeeded in about twenty cases, and failed in two.

I have also used the permanganate in some cutaneous eruptions with good results.

*Ammoniated Tincture of Guiacum in Inflammation of the Throat.*—J. H. Farner, M.D., (Canada Lancet), writes:

Ammoniated tincture of guiacum, in inflamed throats, whether acute or chronic, seems a remedy that is totally unknown to some practitioners, and wholly ignored by others. In fact, the profession seems to have forgotten, to a great degree, that tr. guaiaci ammoniata exists at all. Through your columns, I beg to submit my experience of this remedy for the last quarter of a century, and would ask my brethren in the profession to give it a fair trial, and see if their experience does not fully correspond with mine. "In cases of chronic hoarseness," I use it with almost invariable success in the form of a gargle. A clergyman in this section was always, after preaching, much troubled with hoarseness. He applied to a practitioner of high standing, who excised the uvula. This gave no relief, and he was often, he said, in agony after service. On examining the fauces, the tonsils were enlarged and flabby, the arch, and as far as visible of the fauces was of a deep rusty red, with small white spots. He was very hoarse, and had at intervals spasmodic twitching in the tongue and throat. I gave the following gargle:

| | |
|---|---|
| R.—Tr. Guaiaci Ammon., | dr. iij. |
| Liq. Potas., | dr. iij. |
| Tr. Opii, | dr. ij. |
| Aq. Cinnam., ad. | oz. viij.—M. |

Fiat garg. Utend om. hora.

The liq. pottassa helps greatly to keep the gum dissolved, otherwise it would separate in small black lumps of gum. He was relieved the first time he used it, and in forty-eight hours was perfectly well. In his case the hoarseness was produced by public speaking, and had annoyed him for some years; in fact, so much so, that he told me he would be compelled, from the irritation of the vocal cords, to forego the ministry, if he did not obtain relief; but the use of the gargle perfectly cured him. Others of his profession were also relieved by this prescription.

I use this remedy in inflamed throats, and find it most powerful as a remedy in all sorts of inflammation of the fauces. In the first stages of quinsy its action is astonishing. It seems to scatter the disease at once. Of course if pus has been formed, it cannot be expected to cause absorption, but it will allay the inflammation, and give speedy relief. I do not mean to say that tr. guaiaci, am. will entirely stop inflammatory action in quinsy, but I do say that the irritation and choking sensation are very much relieved.

In cases of inflamed tonsils, and sore throat, when produced by, or occompanying measles, scarlatina, cynanche parotidis and croup, I use the pure tincture, as follows: Tie a bit of sponge or rag on the end of a stick, and saturate it thoroughly, then holding down the root of the tongue with the handle of a tablespoon, I apply the tincture freely to the fauces. This produees a momentary asphyxiation, but when the patient gives a cough it is gone. In general this is repeated every hour or two, according to circumstances. It is common for practitioners to have young ladies to apply them for hoarseness and sore throat, arising from, and in connection with suppressed menstruation. In many cases this will be found a very annoying trouble and difficult to relieve. In such cases, in addition to the above treatment, I use the following:

| | | |
|---|---|---|
| R.—Ferri, Sulph. | oz. j. | |
| Spt. Æther Nitros, | oz. ss. | |
| Morph. Sulph., | gr. iij. | |
| Infus. Gent. ad., | oz. xij. | M. |

Sig.—Coch. mag. ter, quaterve, in die.

The bowels are relieved if necessary, by a few grains of hydr. chlor. and pulv. scam.

In common colds with distressing cough, in whooping-cough, and hectic irritation from phthisis, a gargle of tr. guaiaci. am., although it may not exactly cure, will wonderfully relieve, and is well worthy of trial. If a portion of the gargle passes down the œsophagus, all the better. But the longer it is in actual contact with the irritated fauces, the better will be the result.—

*Thereapeutics of Carbolic Acid.*—This is a potent antiseptic, and is extensively used to correct the fœter of gangrenous and offensive sores. In the treatment of wounds it acts beneficially by averting the tendency to the formation of pus, preventing inflammation, moderating pain, and arresting hæmmorrhage. Mr. J. Wood advocates the employment of sulpho-carbolate of zinc as an application to wounds, suppurating abscesses, after operations, and in gonorrhœa and venereal sores.

*Carbolic Acid* represses exuberant granulations, and acts as a stimulant to weak, indolent and unhealthy ulcers. As a topical application it has been recommended in the treatment of necrosis, caries, carbuncles, diphtheritic surfaces, lupus and cancerous sores. It certainly relieves the pain of cancer, and cases are recorded in which it appeared to have modified the disease. As a gargle in diphtheria, carbolic acid has been extolled, and in other affections of the throat and pharynx, as in ulcerations and enlargement of the follicles, pulverized solutions of it have ordinarily proved beneficial.

Dr. Edgar Browne recommends carbolic acid as an application for sweating feet. In skin affections of crytogamic origin the acid has undoubtedly proved serviceable. In alopecia ereata Dr. Watson uses carbolic acid successfully; he employs a lotion night and morning consisting of one drachm of the acid to three ounces of glycerine; in a case so treated the scalp was covered with abundance of hair in seven months. Dr. Prior, of Bedford, states that carbolic acid will cure porrigo favosa, and that for achorion Schonleinii it is the safest and surest parasiticide. According to Kempster, it effectually destroys acarus scabiei and pedicularis capitis.

*Carbolic Acid* aborts the pustules of cow-pox and small-pox, and a solution of it in oil (1 to 8) is greatly recommended by Dr. Scott, of Dumfries, as an application to prevent the pitting after the latter disease, and also in cases of gunpowder burning.

Anal injections of carbolic acid are of value in mucous diarrhœa of the large bowel, and Rothe recommends the internal administration of it in the treatment of diarrhœa and cholera.

Dr. Fuller and others have found carbolic acid to be of great service in cases of fermentative dyspepsia, even when charcoal has failed to afford relief. It will be found that ten or fifteen grains of sulpho-carbolate of soda taken before food will prevent flatulence occurring after meals.

Carbolic acid is sometimes used for the cure of soft hæmorrhoids, which it effects by corrugating the integument.

In hæmorrhagic ulcers of the stomach the administration of grain doses freely diluted has proved efficacious in checking the bleeding.

Sponging the body with a weak solution of carbolic acid is said to drive away mosquitoes.

In gonorrhœa an injection of twenty grains of sulpho-carbolate of zinc in eight ounces of water, to be used two or three times daily, has proved satisfactory.—*Canada Lancet.*

*Croton Chloral Hydrate in Photophobia.*—This remedy has been used in Guy's Hospital, London, with the following results:

If now we look at the results of these cases, we see that Nos. 1, 3, 4, 5 and 8 were markedbly benefitted by the drug, and No. 6 to a great extent. These cases, Nos. 1, 3, 4, 5 and 8, were the subjects of well marked congenital syphilis. All the cases were the worst I could pick out from among the out patients attending at the hospital. It is right perhaps to state that most of these patients came from miserably dirty homes, where they were probably only half fed; so that the good food and nursing of the hospital may have had something to do with the result.

As regards the dose of the medicine, it will be seen that various quantities were given, the smallest being grs. v. three times a day, and the largest grs. xx., without producing, as far as I could detect, any unpleasant symptom. The only complaint was the exceedingly nasty taste of the drug, which was given combined with syrup of lemons, about dr. iij. to oz. j. of water, in which it was found to be more soluble than in water alone. In only one patient, No. 6, did it cause any feeling of sleepiness. Dr. Bader concluded, from the result of these cases, that it was only in diseases of the eye which were due to syphilis we could expect any marked and permanent benefit.

*Treatment of Chronic Constipation.*—[*The Medical Times and Gazette* contains an interesting article on the "Therapeutics of Chronic Constipation," by Dr. J. K. Spender, of London, and though not of very recent date, we subjoin an extract as presented in the *Half-Yearly Abstract of Medical Science*, relating to the treatment of this annoying malady.] The treatment promises not mere relief, but final cure, and "comprises four therapeutic factors: (*a*) minute and frequent doses of watery extract of aloes, very rarely of extract of colocynth; (*b*) a dose of sulphate iron (gr. jss or ij), always combined with each dose of the direct aperient; (*c*) regulation of the diet; (*d*) constitutional exercise. The author writes chiefly of factors (*a*) and (*b*). The quantity of extract of aloes, in all but extraordinary cases, he says, should not exceed one grain. It is conveniently given in the form of a pill. With this pill there should always be mixed a dose of sulphate of iron, varying from one to three grains; this is the essential point of the treatment. Any other tonic of the neurotic kind cannot supply the place of the iron; iron is not only *facile princeps*, but is not interchangeable by anything else. Extract of nux vomica may be added, if the prescriber pleases, as an ornamental appendage, or as a means of blending the other constituents together; and belladonna is a remedy of definite auxillary power; but both these drugs, *quoad* constipation of the bowels, are uncertain or unsatisfactory, and rarely do permanent good. Dr. Spender begins, then, by desiring adult patient to take a pill composed as above three times a day, immediately after the principal meals.

He is cautioned that at first there will be probably no apparent effect, and that two or even three days may pass before any medical evacuation of the bowels takes place, perhaps even then difficult and discomforting. But within the next forty-eight hours there will be most likely an evacuation of the bowels once or possibly twice in the day; *but nothing approaching to purgation ought ever to be permitted*, and, therefore, the patient must be instructed, on the occurrence of the first loose motion, to withhold a pill, and to take only one in the morning and one in the evening. He then continues for a time his morning and evening pill, and is pleased to discover that so slender a medicament has such a decided effect. Not improbably, at the end of another week or fortnight, he is compelled, by the same reason as before, to drop another pill, and the same result is now brought about by one pill daily, as was originally produced by three pills. Within another month, he may reduce his allowance of medicine to a single pill once or twice a week; and finally his whole scheme of medical treatment becomes merely preventive in its design and scope, and he takes a pill occasionally for the sake of maintaining health and warding off old troubles.

"When there is real or fancied difficulty in the administration of pills, the best way of carrying out the plan above described is by combining the *mistura ferri* with the *decoctum aloes compositum*, the doses being determined by the application of the same principles."

The treatment seems altogether rational, and we hesitate not to recommend it. The object sought is not mere evacuation of the bowels, but restoration of lost tone.—*Med. News, Cin.*

*Treatment of Excoriations of the Os Uteri*—In the Dublin *Journal of Medical Science*, Dr. Halton gives a number of cases, and says on their therapeutics:

The treatment adopted was that which has had its origin in the Dublin School,* and which has, notwithstanding considerable opposition from other quarters—opposition which, it may be remarked, sometimes oversetepped the boundary of politeness or even of pathological good sense—gradually obtained the approval of the majority of the profession. It consisted in reducing local congestion by local means and touching the excoriated surface with the strong nitric acid. This was always carried into the cervix when that appeared diseased, and the acid brought in contact with the whole surface of the canal, and even to the fundus if necessary.† It never gave rise to the

*Ringland.—Kidd. *Dub. Jour. Med. Science*, Feb., 1869.

†It is by no means necessary in all cases to dilate the os before touching the interior of the uterus with nitric acid. In many cases where this becomes necessary, the canal of the cervix is sufficiently patulous to admit the stilette covered with cotton-wool soaked in this agent.

slightest symptom of danger or distress, and in the vast majority of instances was altogether unfelt. When a pain did occur, its amount was so trifling as to attract little notice from either the patient or physician. Astringent injections were found to be of little use, and whether this was from the patient's awkwardness in managing them or not, they have been laterly dispensed with altogether, and their place supplied by the tannin, pessary or bougie, placed in contact with the os or introduced into the canal. The skin of the abdomen has been leeched or blistered, as seemed most suitable, over the tender spot in the region of the ovary, with very marked benefit. When such leucorrhœa was present, small blisters to the sacrum were found serviceable, while ergot and Indian hemp were useful internally, particularly when hemorrhage was present, but, undoubtedly, the most generally affective drugs were strychnine, in small doses, in combination with dilute nitric acid. To these was added some form of tonic, and if local treatment was from any cause inadmissible, this mixture, I think, would afford the best chance of relief. The following is the formula used:

R. Liquor of strychnine... ..............dr. iss
Dilute nitric acid......... ..............dr. ij
Tincture of gentian.....................oz. ss
Hoffman's liquor........................dr. iij
Aqua..............................q. s. oz. viij. M.

The dose is one tabespoonful thrice daily, before meals. If pyrosis is present, which it sometimes is, even in our tea drinking peasantry, a drachm and a half of sedative liquor of opium added to the above for a week or two, taking care to regulate the bowels with suitable aperients, will be found serviceable. In the directions it was not considered advisable to interfere with marital relations, except in case of serious hemorrhage, and while the value of exercise and fresh air was sufficiently impressed, they were enjoined to avoid standing or kneeling as much as possible.

*Bathing in Entero-colitis.*—Dr. C. G. Comegys, of Cincinnati, in the New York *Medical Record,* says: "Before we are called to these cases tentative measures for the relief of the diarrhœa have already been applied by the friends, so that the inflammatory stage is generally fully developed when we first see the patient. The skin is hot (temperature $102\frac{1}{2}°$ to 105°), the pulse rapid (130 to 150), respiration 30 to 59, with frequent purging of semi-fluid, greenish watery, fæcal, and half-digested matters; the mouth and tongue are dry; the thirst intense, but the water taken to slacken it is quickly thrown off; the eyes are staring; pupils contracted; insomnia and rolling of the head, with utterance of distressing cries, due to headache from hyperæmia of cerebral vessels and unappeased thirst. Such is a general statement of the symptoms.

"I at once proceed to give the little sufferer a bath in hydrant water, which with us in summer is about 75°. I have found it necessary to give this my personal attention at first; the mother or friends will not carry out instructions, on account of the cries and resistance of the child; it seems to them a great cruelty. The contact of the hot skin with cold water is certainly painful for the moment, hence I immerse the body from legs upward gradually, sponging the skin in advance, so as to obtain tolerance.

"When the body and extremities are fully under, holding the head in the palm of my left hand, I pour over its surface cooler water, such as cistern water, which is here about 75°. This is kept up for ten or even fifteen minutes. Meanwhile the child ceases to cry or struggle, and is evidently greatly comforted; more especially when cool water is freely given to drink—the greedy swallowing of which shows how much of its distress is due tothirst.

"After the bath the patient should be wrapped, unwiped, in a light woolen shawl and laid upon its bed, with a slight additional covering. The pulse has lost frequency, but is quite feeble; the breathing is slower and the skin quite cool, even bluish in hue. The sedation may seem at first too great, but reaction soon begins, a healthy warmth and perspiration are established, and the child falls into a peaceful sleep. The scene has so changed that one will find no difficulty thenceforth in getting a bath given three or four times in twenty-four hours, if the alarming train of symptoms make show of revival; and they will revive to such an extent as to require exhibitions of the bath from time to time for three or four days perhaps, for the diseased state of the mucous membrane within has not been as suddenly relieved as the abnormal heat of the body.

"In the mean time internal remedies should be freely employed. Quinine, whisky, beef-tea, milk and lime water are the chief agents. One grain of quinine and a dram of whisky every three hours, for a child eight to sixteen months old, looks rather formidable, but they will be found admirable while the disposition to fever lasts.

"Subsequently bismuth and pepsin are of great value to restrain diarrhœa and to assist digestion, so greatly at fault, owing to the blow which the mucous membrane has suffered."

*Treatment of Eclampsia.*—Formerly venesection was resorted to in the most severe cases, but since October, 1868, no patient has been bled, and reliance has been placed on the administration of chloroform, often for many hours consecutively. It has not even been considered a contra-indication to this treatment if the element of coma has preponderated over that of convulsions and there has been stertor and deep lividity of face. A dose of croton oil has also generally been given at the outset. Out of fifty cases recorded before the change in the mode of treatment there were fifteen deaths, or thirty per cent. Out of twenty-three cases reported since there were five deaths, or 21.7 per cent. This latter per centage, however, does not give a fair epresentation of the results of the chloroform treatment, for in two of the fatal cases there was no opportunity for the continuous use of chloroform. In the third the patient, an old epilectic, was not seen till she had been comatose for three days; and in a fourth the fatal issue was perhaps attributed to a too long delay in effecting artificial delivery.

In reference to the treatment by bleeding, it may be noted that in two cases the convulsions came on after considerable post-partum hemorrhage; and that a third hemorrhage occurred after convulsions, and the patient shortly after died unexpectedly from apnœa, the heart's action continuing long after respiration had ceased.

Delivery was effected by forceps in four cases ; in all the rest by the natural efforts. Of the twenty-five patients fifteen were primiparæ, or sixty per cent. In several patients who recovered a modern elevation of temperature was noted, but in only one fatal case was any observation made as to the presence or absence of that hyperpyrexia which is described by Bourneville as generally occurring before death. In this instance the temperature rose to 108.8°. Cold effusion was employed, but too late to be of any avail.—*Louisville Med. News.*

*Antiseptic Treatment of Diphtheria.*—Dr. Post, ((*Medical Times*) referred to a patient whom he was called to visit in the morning, a boy three years of age, who had very considerably swollen tonsils, with diphtheritic patches upon them ; considerable mucous discharge from the nasal cavities ; and grandular enlargement with tenderness at the angle of the jaw. The doctor at once prescribed bromine with bromide of potassium, after the following formula :

R.—Potass. Bromid. dr. i.

Aquæ oz. i. M.

Dissolve in the solution of bromide of potassium, twenty minims of bromine.

Of this mixture administer gtts. x in a teaspoonful of water every hour.

The nasal cavities were hourly washed with a solution of common salt in water, a teaspoonful of salt to the pint of water ; and one grain of sulphate of quinine given in solution every two hours. To these were added a tablespoonful of milk punch every hour.

In the evening the patient was found breathing with much greater ease than in the morning, and was sitting up in bed.

This single case simply showed something for the disinfectant plan of treatment.

Dr. Post also referred to another boy, æt. nine years, who had the disease much worse than the boy just alluded to, and the solution of bromine and bromide of potash was applied by means of a swab, because the patient made no resistance, as was the case in the other patient. In other respects, the treatment was the same. In the latter case also there was marked improvement at the second visit; and at the end of four or five days the patches of diphtheritic exudation had all disappeared, and the patient was fairly convalescent.

Three weeks subsequently, the boy had doubled vision, which was treated by the use of strychnine in 1-60 gr. doses thrice daily, and complete recovery followed.

There was another child of the family at that time, and he was removed from the house. He, however, was attacked with diphtheria a few days afterwards; and, apparently, was no worse than the boy who was under Dr. Post's care, but the case proved fatal.

*Tannin-Dressing for Wounds.*—Dr. Graf, of Elberfield, recommended the use, under proper conditions, of tannin as a disinfectant, in connection with cotton-wool. He is in the habit of covering cotton wadding with a layer of tannin as thick as a knife blade, applying it as a dressing, covered with more batting, and allowing it to remain from four to fourteen days. He succeeds thus in preventing inflammation with its accompanying fever, arresting at once any capillary hæmorrhage, lessening the risk of secondary hæmorrhage, and procuring healing as under a scab. When the dressing is removed, he finds either a more or less complete cicatrix, or a healthy granulating surface without suppuration. He had employed this dressing in over one hundred cases of injury of the hands or fingers by machinery, attended with injury of the bones. In not one of these cases, if brought early under treatment, was there any local traumatic disorder, and fever, if present at all, was very slight. His paper is too long to consider in all its carefully prepared details—*N. Y. Med. Jour.*

*Treatment of Disease of the Stomach by Washing out by Means of the Stomach Pump.*—A novelty in practice, which was suggested by Kussmaul, and introduced in this hospital by Dr. Herzog, consists in washing out the cavity of the stomach by means of appropriate remedies.

The patient upon whom it was practiced was a woman who gave a history of nausea and vomiting. The diagnosis rested between malignant disease and chronic gastric catarrh; but in either case the same treatment was indicated.

The effect of the stomach pump was to make the patient feel easier, so much so, indeed, that it was frequently had recourse to at her request. After a few days the vomiting, which had been nearly constant, became exceptional, and after a short time the patient left hospital very much improved.

In using the stomach pump the method employed was, first to evacuate the stomach, and then inject a weak solution of salicylic acid, which was subsequently pumped out.—*Mount Sinia Hospital, in N. Y. Med. Jour.*.

*Muriate of Ammonia in Diphtheria.*—Dr. E. Sarzana communicates (*Gazz. Med. di Roma*, March, 1876) the results obtained by him in the treatment of diphtheria with muriate of ammonia. He says that he has used this salt on a large scale in this disease, and he has almost always found it efficacious, and regards it as far superior to the other remedies which have been proposed. He has used it externally in the dose of 1–2 (about 20 grains) grammes, powdered on the affected parts, and internally in the dose of 2–5 (about 40 grains) grammes, dissolved in a decoction of cinchona, or in a simple saccharine solution. He states that one or two doses of this salt have caused every trace of the diphtheritic exudation to disappear, and have prevented the appearance of those general disorders which are consecutive to the disease, and that the convalescence has almost always been very rapid.—*Gazz. Med. Ital. Prov. Venete*, April, 1876.

*Choice of Sedatives for the Aged.*—"If we think fit to employ opium as an anodyne or hypnotic with those who have attained to or are on the high road to second childhood, it is judicious to combine chloral and spirit of chloroform with it; the opium being prescribed in excess when pain, the chloral when restlessness, and the spirit of chloroform when cramp predominates; and the quantities of the several ingredients need not be large, as each of them intensifies the effects of the others. The addition of from ten to twenty minims of the tincture of Indian hemp, a very invigorating soporific, to such a mixture as the above, is most serviceable in dealing with a heart enfeebled by advanced age or exhausting illness; and in thus prescribing it I have invariably met with an exemption from the distressing symptoms which sometimes result from the oppressive action of opiates on the respiratory system."—*Dr. Stokoe in Guy's Hospital Reports.*

*The Treatment of Diphtheria.*—On this subject, Dr. William Yeats writes, in the *Edinburgh Medical Journal*, after describing some cases: Regarding the treatment more dependence was placed in the general than in the local. The general treatment was stimulating in the full sense of the term. Nutrition was directed to be administered assiduously night and day. Strong beef-tea, raw eggs beaten up, Liebig's extract of meat, and milk were the principal foods. Port, sherry and champagne, whichever was most agreeable, were given in wineglassful doses every three hours, day and night; and half that quantity to children. The medical treatment consisted of iron and chlorate of potash combined. The iron treatment was introduced by the brothers, Mr. Hamilton Bell, of Edinburgh, and Dr. C. Bell. The form and mode in which it was administered was this: As soon as a case came under my notice, I prescribed, for adults, ten-drop doses of the strong liq. ferri perchloridi, with ten or fifteen-grain doses of chlorate of potash, to be taken in a wine-glassful of water every two hours, until decided improvement was evident, when the interval was prolonged. The dose was modified for children, of course. Vomiting was usually set up by the first two or three doses, but the patient, being warned of this, was instructed to persevere, and afterward there was no trouble in this way. Canstipation was rarely complained of, and when it did happen, a little glycerine added to each dose obviated the difficulty. The local treatment, which at first was employed, consisted of a strong solution of iodine, viz:

| | |
|---|---|
| R.—Iodine, | gr.xlviij. |
| Potassii iodidi, | gr.xxiv. |
| Sp. vini rectificati, | dr.j. |

This was painted on the affected parts of the throat daily, and seemed to be very useful. But finding the internal exhibition of the iron so satisfactory, I adopted its local application also, in the following form: Liq. ferri perchloridi and glycerine, in equal parts, which was brushed over the exudations daily. The glycerine helps to dissolve the slough, and the iron, I think, hardens the subjacent surface, and considerably modifies further exudation. Whenever laryngeal symptoms were observed, the patient was immersed in a warm bath for five or six minutes, then wrapped, undried, in a warm blanket, to induce free action of the skin, which was kept up by hot water bottles placed about it. This was immediately followed by an emetic of sulphate of copper in five-grain doses to children, which usually induced free vomiting in the course of five minutes; the throat was then well swabbed with either of the local applications, and very soon the most harassing symptoms were relieved. If there was any return the same treatment was repeated. The results were very gratifying, indeed. In one case vomiting could not be excited by any means; but the strength, on the other hand, was well maintained, and the child recovered after two week's illness.

*Carbolic Acid in Carbuncles.*—Dr. Peter Eade writes to the *British Medical Journal:*

I believe it to be general experience that the pimple in which a boil begins its life and career may be destroyed by any common caustic, if thoroughly applied. I venture to assert also that a carbuncle, even when very considerably advanced and of very considerable size, may in like manner be destroyed by the free application of carbolic acid to its centre and other parts.

The essentials for its proper action, so far as my experience has gone, appear to be these:

1. The acid must be applied in *strong* solution (four or five parts of acid to one of glycerine is the strength I employ.)

2. It must be brought into contact with the diseased tissue, for it appears to exert no influence on or through the unbroken skin. To this end, if sufficient opening do not exist when the case is first seen, a proper one must be fearlessly made in the very centre of the disease by some appropriate caustic, and, perhaps, the acid nitrate of mercury effects this better and with less discomfort than any other.

3. The acid solution must be occasionally reapplied to, and into, the hole thus formed, or those already existing, and I have found it a good plan to keep a piece of lint wet with a weaker solution constantly over the sore.

Take the following example, which has occurred to me within the last two or three weeks:

A lady, aged 40, showed me a boil on the left buttock, of six days' duration. It was circular, with a diameter of four inches: was red and angry-looking; tender, hard at its base, and rapidly increasing. To the prominent point in its centre I freely applied acid nitrate of mercury over a space of about one-third of an inch in diameter. Next morning, I removed the scab which had formed, and freely passed the strong carbolic solution into the little opening formed in the mass, as well as I could, with a quill pen charged with the liquid (and I may say that I find this a very convenient instrument for the purpose.) At this time the swelling had increased considerably in size, was more tender and inflamed and painful, and was threatening to be a very formidable case of the disease. Now mark the effect of the treatment. The acid was freely applied twice more, during the day, and the very next morning, on my visit, it presented the appearance of having suddenly collapsed. It had shrunk greatly in size, was flabby, and far less painful, and its vitality was plainly destroyed. In four or five days nothing remained but a little hardness about its base, and it rapidly got quite well. No core was ever discharged, and no pus appeared after the first application of the carbolic acid.

*Local Treatment of Burns.*—In an article in No. 226 of the *Medical Times*, on "The Pathology of Burns and Their Rational Treatment," by Bedford Brown, M.D., of Alexandria, Va., the writer, under the latter head, says:

"Of all local applications in the experience of the writer, iodoform, prepared with extract of conium, and spermaceti ointment, with a small portion of carbolic acid, appears to meet the several indications best.

"This agent acts as a certain and most effective sedative on the painful and irritable exposed surface, and at the same time as an antiseptic. It reduces irritation, inflammation, and suppuration when in excess, in a remarkable manner. It converts a most painful and irritable wound into one comparatively painless, with promptness.

"This remedy is also an excellent promotive of healthy action and of the healing process. I have experimented with iodoform ointment in these cases repeatedly, and always with the same pleafant result. The use of this preparation has another advantage; it renders the constant use of anodynes unnecessary. The following formula has been found the best;

R. Iodofoami ................................dr. ij.
Unguent. cetacei ..........................oz. i.
Ext. Conii ...................................dr. iss.
Acid. carbol ................................ gtt. x.
M.

"This ointment is spread twice daily on soft linen, and applied over the inflamed surface, and then enveloped in oiled silk. No other dressing is necessary. The only objection to the use of this ointmcnt is its peculiar odor. In those cases of burns attended with great *dryness* of surface from destruction of vitality and want of exhalation, the wound, before bsing covered with the iodoform ointment, should be coated over with the common linimentum calcis. This affords a soft and moist dressing, which in no wise interferes with the action of the iodoform."

*Fibroid Tumor of the Prostrate Successfully Treated by Injection of Iodine.*—(*Virginia Medical Monthly*, June, 1876.)—Dr. Melville Taylor reports the case of a man, æt. 26, who, when he first came under servation, had the following history: About nine months previously he had discovered a tumor the size of a chestnut in the perineum, just behind the scrotum; it was at first movable, but soon became stationary. Its growth was progressive. He had never had any pain, but complained of a sense of weight and dragging in the perineum, and of severe tenesmus. He urinated frequently, slowly, and with much straining; the water at times containing mucous, and being ammonical. Latey his urine had been dribbing from him. His walking was greatly interferred with by the tumor between the thighs, and it was for this reason only that he applied for relief.

Exploration of the prostrate by rectal touch revealed an abnormal enlargement of this organ. It was hard and firm, presenting to the fingers four different segments. No increased sensibility. Upon the passage of the catheter, an obstruction was met with at the prostatic portion of the urethra; but this, after some manipulation and not a little pain to the patient, was overcome, and the instrument slipped into the bladder, when about oz. xx of fetid urine was passed, although he had urinated previous to its passage. The catheter caused some pain when impigned against the walls of the bladder.

The diagnosis of fibroid being made after a few other examinations, treatment was commenced by the injection of iodine into the tumor, fifteen drops of the tincture being used at intervals of several days. There was some little irritation at first, but this soon subsided, and the final result was a complete cure, the prostrate decreasing from the size of a base ball to its normal dimensions.

*Treatment of Cystitis by Atropia in enemata*, (*Virginia Medical Monthly*, June, 1876).—Dr. S. W. Semple reports four cases of acute cystitis, out of a large number which he has successfully treated by a method which is not very commonly employed. It consists in the administration by enema into the rectum of from minims xl to dr. j of a solultion of sulphate of atropia (gr. j to water oz. viij), to which is added sufficient carbolic acid to prevent the formation of organic entities and the deposit of the atropia The dose is added to oz. ss of water for administration, and given twice in twenty-four hours. It uniformly and immediately arrests the frequent strangury and the painful micturition, gradually checks the mucous and sanguineous discharges, and relieves the suprapubic pain with the cystic inflammation. When the urine is alkaline, Mettauer's nitromuriatic acid is given to correct it; and when it is so acid as to irritate, the acidity is corrected by antacid remedies, of which the bi-carbonate of potassium with subnitrate of bismuth is generally preferred, because of the tonic effect of the bismuth and its very soothing effect on the mucous surfaces of the urinary organs. When constipation exists, which is frequent, it is relieved as occasioan requires, generally by the German pulveris glycyrrhizæ compositus, until the bowels begin to act regularly from the effect of the atropia, which generally soon results.

*Absorption of Iodine by the Skin.*—M. Jules Simon reports a number of cases of the absorption of iodine followed by albuminuria in children.

Applications of equal parts of glycerine and tincture of iodine were made each day in the treatment of a number of cases of favus. The results obtained were quite satisfactory, until manifestations of iodism were noticed in a child of ten years (papular eruption, congestion of the nostrils, epistaxis, thirst). Albumen was found in urine, and iodine. An examination of the urine of all the cases treated by the external application of iodine proved that there was albuminuria in more than half the cases. The albumen disappeared when the iodine application was stopped, but returned when the medicament was again used. In two cases of phthisis, and in one of white swelling of the knee, albumen was found in urine soon after tincture of iodine had been locally applied. Further observations proved that painting over a limited area caused no albuminuria ; if, however, more than ten centimetres square was covered, albuminuria resulted in many cases.—*Gaz. Hebdom.*, May 12, 1876.

*Ulcerated Nipples.*—M. Legroux advises the following treatment : Spread with a camel hair brush a layer of elastic collodion around the nipple, in a radius of an inch or more ; a piece of gold beater's skin should then be paced over the nipple and collodion, taking care to make a few holes with a pin over the part of the gold beater's skin which covers the nipple, so as to allow the milk to ooze through. No collodion should be spread on the nipple itself, as some pain might thereby be occasioned. By the rapid evaporation of the ether the collodion dries up, and the gold beater's skin adheres. The nipple is then more or less pressed down by the latter, which in drying becomes tense. When the child is to be nursed, the end of the nipple should be wetted with a little water. The gold beater's skin which covers it becomes soft and supple, allows the nipple to swell, and protects the ulcers and fissures from the strain of suction. The mother or wet nurse thus suffers no pain, and the ulcers heal in a few days.—*Lancet*, Dec. 11, from *Aunales de Gynecol·ogie*, Nov. 1875.

*Advances iu Gynæcology During the Year* 1875.—Dr. P. F. Mundi (*American Journal Obstetrics*, April, 1876) concludes an admirable report upon the progress of gynæcology during 1875 as follows:

1. Among the subjects which the past year has witnessed an advance is that of the normal structure of the uterine mucosa before, during and after menstruation, the nature of menstruation, and its relation to ovulation.

2. The treatment of uterine fibroids by ergot, enucleation, traction, above all by gastro-hysterotomy, the percentage of recoveries from which operation (six recoveries out of nine opertions) certainly seems to promise a bright future for this still much dreaded measure.

3. The vesico-vaginal and vesico-rectal touch, and the daily increasing popularity of the method of treating diseases of the female urethra and bladder by dilatation of the urethra and local applications.

4. The growing appreciation of the influence of posture on the health of the female sex, and the recognition of the value of posture, especiallly if aided by pneumatic pressure in the treatment of uterine displacements.

*Raw Onion as a Diuretic.*—Dr. G. W. Balfour, (*Edinburgh Med. Jour.*, September, 1875) records three cases in which much benefit was aflorded patients by the eating of raw onions in large quantities. They acted as a diuretic in each instance. Case first was a woman who had suffered from a large white kidney and constriction of the mitral valve. Her abdomen and legs had been tapped several times, but after using onions as above she had been free from dropsy for two years, although still suffering from albuminuria. Case second suffered from cardiac disease cirrhotic liver and ascites. Case third had ascites depending on tumor of the liver. In both of them the remedy had been used with good results. Both had been previously tapped, purgatives and diuretics alike having failed to give relief. All other treatment having failed to give relief, recourse was had to the onions. Under their use the amount passed steadily rose from ten to fifteen ounces to seventy-eight or a hundred.

*Chlorate of Potash in Infantile Diarrhœa.*—Dr. Moncorvo de Figueiredo, in an article in the *Revista Medica de Rio de Janeiro*, narrates the circumstances which led him to appreciate the efficacy of chlorate of potash in a case of obstinate diarrhœa, lasting eight months, in a child two years of age. A solution of the potash, which had becn ordered for some other case of throat trouble, was given to this child by mistake. The effect was, however, very beneficial; so much so, that Dr. Moncorvo directed the medicine to be continued, and he soon obtained a complete cure.

Dr. C. Bonfigli, in the Italian journal, *Il Monimento*, also extols the beneficial effects of this salt in cases of diarrhœa, which he calls *vaso paralitica* of cachectic children. He refers to fifteen cases in which the chlorate was used very successfully, in doses varying between two and ten grammes in the twenty-four hours. —*Gazeto Medica da Bahia*, January, 1876.

*Citrate of Quinoidine.*—From researches orignally made by Buchner, and recently repeated by Haller, in the General Hospital at Vienna, citrate of quinoidine is as certain a cure for fever as the ordinary sulphate, which is ten times more expensive. [Does he not refer to the sulphate of quinine ?] Haller gave the solution of one drachm of citrate of quinoidine in three ounces of water, with the addition of half an ounce of aquæ cinnamoni vinosa in the intermissions, at intervals of from two to three hours, in lieu of spoonful doses. Of forty-one cases of intermittent fever, forty were cured with the solution of quinoidine, and only one relapse occurred, which was again cured by persisting a little longer with the use of the citrate.—*The Practitioner*, from *Neues Rep. d. Pharm.*

R. Acidi carbolic.............................scrup ;
Syrupi lemonis.................................oz. iv.

M. S. A teaspoonful three or four times a day. In Hooping-Cough.

*Chloroform in the Surgery of Infants.* — Dr. Bergeron, of Paris, (*London Obstetrical Journal*) from his personal experience upon this subject, concludes that—

1. Chloroform in the infant is endowed with an almost absolute harmlessness.

2. The child, not having come to the age of reason, nor feeling any moral emotion, suffers from no apprehension of the dangers to which it may be exposed, nor experiences the apnœa which produces so much terror, and is, the author imagines, a most important cause of death supervening suddenly during the administration of chloroform.

3. Chloroform may be administered to the infant from the first day of its birth.

*Traumatic Tetanus in the Horse.*—Mr. R. W. Finlay, V.S., of this city, sends us a report of two cases of traumatic tetanus in the horse cured by the following ;

R. Potass bromid..............................dr. iv.
Chloral hydrat..............................dr. ij.
Aquæ........ ..............................oz. viij.

M. Fiat Sol.

S. To be taken in one dose and repeated three times daily.—*N. Y. Med. Rec.*

*How to Make Leeches Bite.*—The *Le Progres Medical* advises, in order to make a leech bitte readily, to place it in a glass containing cold water, to wash the skin of the patient with warm water, and apply the glass at once.

*Prophylactic in Enlarged Tonsils.*—In a paper in the American *Journal of Obstetrics*, Dr. Robinson states that in the prophylactic treatment of chronic enlargement of the tonsils in children, he attaches great importance to the habit of *cold* bathing. The feet should be kept warm and dry, and he thinks the following rules should be impressed on all mothers and nurses in charge of children : 1. Not to keep the nursery at too high temperature, and always to have a basin, pitcher or bucketful of fresh, pure water, with the surface exposed to the air, contained in the room. 2. Not to allow children to approach a fireplace or register immediately upon entering or leaving a house. 3. Not to keep extra wraps on their little charges, when at home, a moment longer than is absolutely essential.—*Med. and Surg. Rep.*

*Conium.*—Conium may be considered the regulator of dis turbed voluntary muscular movement, and it is especially indicated in diseases accompanied by clonic spasm the result of direct or reflex irritation of the cerebro-spinal motor tract, and requires to be given in such strength as to develop its paralyzing effect upon these centers. But when the spasm is limited in duration, recurrent or periodical in outbreak, as in laryngismus stridulus and other convulsive disorders of childhood, in epilepsy at all ages, and in puerperal eclampsia, the sedative action (properly so called) of chloroform-inhalation will be brought into play with more advantage, as it is more rapid, direct and transitory, and its administration is practicable during insensibility or amid struggles for breath.—*Dr. Stokoe in Guy's Hospital Reports.*

*Glycerol of Subacetate of Lead* is recommended by Balmanno Squire as a very useful application in chronic eczema and other skin diseases. It is prepared of the same strength as the liquor plumbi subacetatis, substituting glycerin for the water. The heating is effected in an oil bath, care being taken to keep the temperature a little below the boiling point of glycerin, and when the reaction has been accomplished, the solution is to be filtered while hot, since after cooling it is too viscid to pass through the filter. For use it is diluted with from three to seven times its quantity of glycerin, as occasion requires.—*Phar. Jour. and Trans.*

*The Penitentiary for a Vendor of Adulterated Milk.*—A milkman here has recently been sentenced to one month in the penitentiary for selling adulterated milk; and, having contested the power of the Board of Health to institute the prosecution in such a case, the sentence passed upon him at the Special Sessions has now been affirmed by the General Term of the Supreme Court. This decision is important, being the first case of the kind carried to the Supreme Court. The power of the Board of Health to act in such cases had previously been undetermined, and there was some apprehension of a judgment adverse to it.—*Pacific Med. & Surg. Journal.*

*Treatment of Diphtheria.*—Dr. S. H. Moore, of Syracuse, N. Y., claims considerable success in the treatment of diphtheria, by the internal administration of iodine. He uses the following:

R. Tinct. iodine ..............................dr. iv
Sacch. alb ..............................oz. iij
Aquæ ..............................oz. iv
M. Cap. dr. i. q. ½ hor.

He writes: "I had during January and February, thirteen cases, and in all of them I pursued the same plan of treatment. All the cases turned out successful, not one dying. The effect of the iodine seemed to prevent the formation of the membrane. It also prevented the swelling of the glands of the throat, and seemed to stimulate the patients, as in no one of the cases cited was there the great loss of strength that we usually find in diphtheria. The above formula is pleasant to the taste and can be retained on the most delicate stomach. The mixture, at first, is of the color of iodine, but during from six to twelve hours becomes almost as transparent as water."

*Treatment of Nasal Catarrh by Nitrate of Bismuth.*—A letter from London to the Phila. *Med. Times* says that the newest thing there in therapeutics is the plan of treating nasal catarrh by the insufflation of bismuth, advocated by Dr. Farrier. He first tried it in his own case, taking a pinch from time to time, and was speedily cured. His further experience decided in favor of an admixture of gum acacia in powder, and the addition of a little morphia. Another new thing is an ornamental bottle, containing a little piece of lint at one end and some nitrate of amyl in the other compartment, for the relief of palpitation of the heart, hysterical or gouty—*Pacific Med. & Surg. Journal.*

*Phosphorus Pills.*—Messrs. Allen and Hanburys suggest the following formula as an efficient substitute for that of the British "Pharmacopœia," which combines the phosphorus with tolu balsam and yellow wax: 2 grains of phosphorus dissolved in sufficient bisulphide of carbon, are mixed with powdered soap and guaiac resin, of each gr. xxxv, glycerin gtt. xii and powdered liquorice root gr. xii or sufficient to make a mass weighing gr. c. The bisulphide evaporates readily, and the mass formed is of good consistence, easily manipulated, readily miscible with other remedies, and what is most important readily soluble.—*Phar. Jour. and Trans.*

*Pancreatic Meat Emulsion* has been brought to the notice of the Berlin Apothecaries' Society, by F. Riedel, who obtained from Dr. Rosenthal the following foımula for its preparation: 250 grams of finely scraped beef are triturated in a mortar with 25 grams of pancreatic liquid (from the hog) and warm water, until a homogenous, light redish brown emulsion-like mixture results, which is injected per annum with some pressure, the bowels having been previously cleansed by warm water injection. It is stated that persons have thus been nourished, who for months were unable to swallow or digest food taken in the ordinary way.—*Phar. Zeit.*

*Formula for Diphtheria.*—

R. Potas. chlor........................................oz. j.
Tinct. capsicum...................................f. oz. iij.
Liq. ferri perchlor................................f. oz. j.
Dilute alcohol }
Pure water, } *aa*...........................f. oz. vj.

SIG.—A teaspoonful in a wine-glassful of water, to be used as a gargle each time. After gargling, a teaspoonful in a tablespoonful of water, more or less, to be slowly swallowed, and repeated every three to six hours.—*Canada Lancet.*

*The Preservation of Raw Meat* is effected by A. Herzen by immersing it for 24 or 36 hours in a solution containing 150 boric acid, 30 borax, 15 table salt and five saltpetre in 2,000 parts of water. The meat retains its fresh appearance and may afterwards be packed in barrels.—*Chem. Centralbl.*, 1876.

R. Magnesiæ sulph..............................oz. ss;
Morphiæ sulph.................................grs. ss;
Aquæ.................................................oz. iv.
M. S. A teaspoonful every half hour. In simple Dysentery.

